# Lysteria Monocytogenes Infection during Monochorionic Twin Pregnancy: Case Report and Review of the Literature

**DOI:** 10.3390/jcm13206061

**Published:** 2024-10-11

**Authors:** Sofia Roero, Chiara Peila, Silvana Arduino, Sonia Deantoni, Alessandra Coscia, Alberto Revelli

**Affiliations:** 1 Twin Pregnancy Unit, Gynecology and Obstetrics 2U, A.O.U. Città della Salute e della Scienza, Sant’Anna Hospital, University of Turin, 10126 Turin, Italy; 2 Neonatal Unit, Department of Public Health and Pediatrics, University of Turin, 10126 Turin, Italy

**Keywords:** listeriosis, pregnancy, twins, neonatal care

## Abstract

Listeriosis is a rare but severe foodborne disease caused by *Listeria Monocytogenes* (LM), a small facultative intracellular bacillus. When occurring in pregnant women, it can be vertically transmitted to the fetus and the newborn. Infected women usually display aspecific and mild symptoms, and rarely develop the severe forms of the disease (such as neurolisteriosis). On the contrary, fetal and neonatal listeriosis can lead to complications such as fetal loss, preterm birth, neonatal sepsis, and respiratory distress syndrome (RDS). Prompt diagnosis is one of the main challenges because of the aspecific presentation of the disease; therapy relies on antibiotics that reach high intracellular concentration and can penetrate and pass the placenta reaching the fetus. Herein we report an infrequent case of LM infection involving a woman with monochorionic diamniotic twin pregnancy, followed by a comprehensive review of the available literature on listeriosis in pregnancy.

## 1. Introduction

Listeriosis is a rare and potentially severe foodborne disease caused by *Listeria monocytogenes* (LM), a Gram-positive, motile, facultative intracellular bacillus. In 2022, 2728 confirmed cases of LM infection were signaled in the European Union (EU) [1]; the EU/EEA reported rate was 0.62/100,000 individuals, slightly higher than the previous year. These cases resulted in 1330 hospitalizations and 286 deaths [1]. Listeriosis was the fifth most frequent human zoonosis in the EU and one of the most serious foodborne diseases under EU surveillance [1]. Germany, France, Spain, and Italy had the highest number of cases (548, 451, 437, and 345, respectively), corresponding to 54.5% of all European cases [1]. Denmark and Finland had the highest rates (1.5 and 1.3/100,000, respectively). Of note, the incidence of LM infection among pregnant women is usually much higher than in the general population, being around 12/100,000 pregnant women [2].

LM is ubiquitous in nature and has a high resistance: it can survive and replicate in a wide range of temperatures, from 0 to 45 °C. Thus, this bacillus can contaminate a large amount of processed and unprocessed food products of animal or vegetable origin [2]. The main goods at risk for LM contamination are ready-to-eat food and products with a long shelf-life: smoked salmon, meat products (hot-dogs, processed meats), cheese, unpasteurized milk, and frozen vegetables [3,4,5].

In healthy adults, listeriosis can present as a self-limiting gastroenteritis, with fever, diarrhea, nausea, and vomiting occurring in the first seven days after exposure; the severe form with bacteriemia and central nervous system infection (neurolisteriosis) mainly occurs in immunocompromised or elderly individuals.

LM infection may occur in pregnant women and is defined by the presence of LM in any sample of maternal, fetal, or neonatal origin [1,2]. LM infection during pregnancy is generally mild: a prospective cohort study showed no evidence of severe sepsis, neurolisteriosis, or death in infected mothers; however, only 5% of pregnant women with listeriosis had normal delivery and post-partum evolution [6]. The same cohort study showed indeed that 70% of the neonates born from infected mothers had abnormal clinical status at birth, including respiratory distress syndrome (RDS); 70% developed early-onset sepsis and 6% late onset sepsis, many of them presenting with acute meningitis; finally, 3% died, 6% had severe brain injury, and 2% had severe bronchopulmonary syndrome [7].

## 2. Case Report

During a routine prenatal care assessment, a 33-year-old woman with monochorionic diamniotic twin pregnancy at 30 weeks and 6 days of gestational age was found to have reduced cervical length (16 mm) with funnel. The pelvic exam found a 2 cm dilated cervix; considering her four prior vaginal deliveries and the irregular uterine contractions, the patient was immediately hospitalized. At admission, cervical–vaginal swab and urine culture were performed. Maternal anemia (Hb 9.5 g/dL) was discovered and treated with intravenous ferric carboxymaltose (1000 mg).

During the second day of hospitalization, increasing uterine contractions appeared, and tocolytic treatment (Atosiban plus Magnesium Sulfate) was administered to allow a complete course of antenatal corticosteroids for fetal lung maturation in order to prevent respiratory distress syndrome. No changes in the cervix were detected in the pelvic exam.

The following day, fever appeared (37.7 °C), which soon increased to 38.4 °C: paracetamol and empiric antibiotic therapy with Imipenem were started after collecting blood samples for culture. A chest X-ray was performed and resulted normal, while blood exams showed increased C-reactive protein (CRP; 60 mg/dL), with negative procalcitonin.

After two days from the onset of the fever, CRP increased to 92 mg/L and blood culture resulted positive for LM; also, an antibiogram for susceptibility testing was performed. Antibiotic therapy was switched to Ampicillin (1.5 g every 6 h) plus Gentamicin (80 mg every 6 h). Vaginal swab and urine culture were negative. The fever disappeared completely after 3 days of targeted antibiotic therapy. After 6 days, Gentamicin was discontinued, while Ampicillin was switched to 3 g every 6 h. No other specific symptoms were reported by the patient.

In total, antibiotic therapy was carried out for 21 days. CRP gradually decreased until returning to within normal ranges. Five days after discontinuing the antibiotic therapy, the woman had a diffuse itching cutaneous rash, which required antihistaminic administration. The symptom spontaneously regressed shortly after.

During the hospital stay, intensive fetal monitoring with cardiotocography was performed three times a day, coupled with doppler evaluation of uterine and umbilical arteries twice a week.

Detailed counseling regarding the risk of maternal and fetal sepsis was offered to the patient, with the participation of neonatologists, and thorough information regarding the importance of antibiotic therapy was given.

After two weeks of hospitalization, ultrasound assessment of fetal biometry detected intrauterine growth restriction (reduction in abdominal circumference and femural length) for the first twin, whose doppler velocimetry of umbilical artery resulted abnormal (pulsatility index >95th centile), with preserved end-diastolic flow. The second twin had normal growth and umbilical artery doppler velocimetry. No sign of twin-to-twin transfusion syndrome or twin anemia–polycythemia sequence emerged.

After more than one month since hospital admission, at 35 weeks and 2 days of gestational age, the patient went into spontaneous labor. The first twin, E., was born at 4:15 a.m. by spontaneous vaginal delivery, with an APGAR of 9/9 and a birthweight of 2120 g (15° centile) and a head circumference of 31.4 cm (18°). Umbilical artery blood gas showed a pH of 7.276, a base excess of 4.5, and blood lactate levels of 4.3 mmol/L. He had a hospital stay of 16 days, during which he was treated for mild hypoglycemia shortly after birth, presented jaundice that required a single course of phototherapy, and was treated with prophylactic caffeine citrate; he never showed any signs of infection (neither clinical nor at laboratory tests) and blood culture resulted negative. A cranial ultrasound was performed and resulted normal.

The second twin, S., however, despite being cephalic at the beginning of the labor, ended up in a transverse position with a shoulder presentation and cesarean section became necessary: he was born at 04:35 a.m., with an APGAR of 6/8, a birth weight of 2490 g (46° centile), a head circumference of 33.7 cm (77° centile), and required CPAP and oxygen therapy up to FiO2 0.3 at birth. Umbilical artery blood gas showed a pH of 7.176, a base excess of −7.2, and blood lactate levels of 6.8 mmol/L. Shortly after birth, S. developed a respiratory distress syndrome which required CPAP and oxygen therapy up to FiO2 0.3 during the resuscitation. During the first few days of hospitalization, due to the worsening of his general conditions and the increasing oxygen requirement and CRP level, S. was first started on antibiotics (after collecting blood samples for culture) with Ampicillin/Sulbactam 50 mg/kg twice a day and Amikacin 15 mg/kg/day and then intubated while surfactant therapy was administered at the standard dose of 200 mg/kg. Due to the onset of neurological symptoms (marked irritability, predominance of extensor tone and opisthotonos), an electroencephalogram (resulting normal) and a lumbar puncture were performed on the following day. Blood cultures (both at birth and on day 2 of life) and cerebrospinal fluid culture and microarray all resulted negative.

S. was extubated after two days and was then assisted with non-invasive respiratory support for a total of 20 days, with oxygen requirement progressively decreasing. CRP levels reached their maximum on day 2 of life (31 mg/dL), then rapidly decreased and normalized on day 8 of life; antibiotic therapy was discontinued after 5 days for aminoglycoside and after 7 days for Ampicillin/Sulbactam. Cranial ultrasound initially demonstrated minimal vasculitis, rapidly resolved by the time of hospital discharge. An echocardiogram, performed due to the worsening of respiratory conditions, detected the presence of a small interatrial defect and a patent foramen ovale. He was discharged home after 24 days in the intensive care unit. Both twins had a favorable evolution during the following six months, without complications up to the current point.

Macroscopic histological examination of the placenta showed signs of malperfusion on both maternal and fetal sides. Also, there were signs of partial placental abruption with different onset times.

## 3. Literature Review

### 3.1. Physiopathology and Diagnosis of Listeriosis in Pregnancy

Listeriosis is a foodborne illness. *Listeria Monocytogenes* enters the gastroenteric tract and is able to cross the intestinal epithelium barrier without disrupting the membrane integrity, hence avoiding being exposed to antibodies or neutrophils [8]: infection spreads after binding to E-cadherin, a cell-to-cell junction protein located in the basolateral membrane of the host enterocytes [9,10]. After crossing the intestinal epithelium barrier, LM invades the mesenteric lymph nodes and enters the bloodstream (primary bacteremia). Consequently, LM disseminates to the liver and spleen, where it starts to replicate. In immunocompetent individuals, the infection is promptly cleared by polymorphonuclear cells and remains subclinical; when it cannot be limited, as occurs in elderly or immunodeficient individuals, after hepatocyte lysis the bacillus is released in the bloodstream and a secondary bacteremia develops [11].

In pregnant women, LM has the ability to adhere to the trophoblastic epithelium and invade the trophoblast layer, accessing the core of the placental villi; junctions at the villus level seem to be the main site of invasion [9,10,12,13]. The placenta thus becomes a reservoir for maternal reinfection [14], and placental micro-abscesses may be histologically evident [15]. Vertical transmission to the fetus is possible and, in the first weeks of pregnancy, it can cause severe malformations because of organogenesis disruption.

The highest incidence of maternal listeriosis tends to occur in the last weeks of pregnancy: the most likely pathophysiological explanation for this phenomenon is the fact that syncytiotrophoblast is one of the main sites of invasion [6,7]. However, it should also be considered that listeriosis in the first weeks of pregnancy tends to result in miscarriage, in which case no culture is made, and thus could be underdiagnosed [7].

Diagnosis is made by isolation of LM in blood, cerebrospinal fluid, or, more rarely, joint fluid. Listeriosis in pregnancy is usually diagnosed by a positive blood culture; after delivery, it can also be diagnosed via the placenta or the newborn’s gastric fluid. The prospective MONALISA cohort study reported the highest sensitivity for placenta and newborn gastric fluid (both resulted positive in 78% of samples); blood cultures were positive in 55% of samples [6]. Anyway, in clinical practice, the great majority (98%) of diagnoses are made from maternal or placental samples [6]. Maternal stool cultures are not useful in the diagnosis of listeriosis because accidental ingestion of the bacterium is quite common, and intermittent fecal carriage and shedding of Listeria is not indicative of infection [16]. Vaginal swabs are also generally not useful in the diagnosis, reflecting the hematogenous seeding of the placenta [2,11,17].

Other than isolation from cultures, attention should be focused on blood tests: usually, symptoms are associated with leukocytosis and elevated CRP, with negative procalcitonin [6]. On wet mount microscopy, the bacillus may be seen in characteristic “tumbling” motion [18]; it may also be evident at Gram staining, particularly if performed on neonatal meconium before polymicrobial colonization of the gut.

### 3.2. Symptoms of Maternal Listeriosis in Pregnancy

Diagnosis can be difficult because there are no specific symptoms, and presentation may be mild: more than two-thirds of LM-infected pregnant women have fever associated with contractions, preterm labor, or abnormal fetal heart rate [6,11,18]. Other symptoms include vomiting or diarrhea, flu-like symptoms (muscle and joint pains, chills), back pain, headache, and sore throat [6,18]. About one-third of women are asymptomatic [16,18].

Pregnant women very rarely develop severe sepsis or neurolisteriosis [19]; no case of maternal death was recorded in the MONALISA cohort [6]. However, only about 5% of infected women have normal gestation, delivery, and puerperium [6]. The rate of cesarean section among infected women is also quite high (40%), mainly due to an increased incidence of abnormal fetal heartbeat at the non-stress test [6].

### 3.3. Fetal Listeriosis

Fetal LM infection may occur by direct hematogenous transplacental route, which is the main pathway, or, more rarely, via inhalation and ingestion of infected amniotic fluid.

Fetal outcome is strictly dependent on gestational age at the time of infection, with the early infection being at higher risk for miscarriage or stillbirth [20,21,22,23]. The MONALISA study identified a cutoff of 29 weeks, after which the risk of fetal death becomes minimal; this adverse outcome usually occurs before admission to the hospital or within the first 2 days of hospital stay [6]. However, fetal demise occurs overall in about one-fourth of infected mothers and it is more common than neonatal death [6,18,21]. A higher proportion of cases without maternal symptoms result in live birth compared to cases with symptoms [23].

The risk of preterm delivery is reported to be about 50%, with fair consistency among available studies [6,18,20,24,25]. As might be expected, perinatal outcomes are worse in case of birth before 32 weeks of gestation, which occurs in 42% of cases; only 15% of premature births take place between 34 and 36 weeks [6].

About one-third of fetuses with infected mothers show signs of fetal distress [24]; meconium amniotic fluid has been found in 21–75% of cases [6,24,26]. Increased risk of early premature rupture of membranes (pPROM), chorioamnionitis, and vaginal operative delivery has also been reported [23,26,27].

Overall, according to the MONALISA cohort, listeriosis in pregnancy has a relevant impact on fetal outcome, with 83% of infected women displaying major fetal adverse outcomes including fetal death, severe prematurity (<32 weeks of gestation), or early or late onset neonatal listeriosis [6].

### 3.4. Neonatal Listeriosis

About 15% of newborns from LM-infected mothers are uninfected, with normal development [6,28]. Neonatal listeriosis is caused by vertical transmission, which may occur via the hematogenous transplacental route, by inhalation of infected amniotic fluid, or, quite rarely, by ascending colonization from the vagina [28].

The incidence of neonatal listeriosis is reported to be approximately 8/100,000 live births [29,30]. The mortality rate for neonatal listeriosis is very high, reaching 24% of infected neonates [20]; however, a recent prospective study reporting data on neonates from the MONALISA cohort suggested that it might be as low as 3% if precocious diagnosis and initiation of treatment are obtained [7]. Infants can develop early-onset listeriosis, mostly with symptoms arising at birth or within the first 24 h, or late-onset listeriosis (within 7–22 days of life). The majority of newborns (70%), usually born from a symptomatic mother, develop early-onset listeriosis, whose presentation is quite similar to that of group B Streptococcal (GBS) infection [18,28].

It mostly presents with acute respiratory distress syndrome and can be sometimes associated with neurological symptoms (lethargy and/or altered consciousness and/or seizures) or cardiocirculatory impairment [7]. These symptoms may represent nonspecific signs of sepsis, pneumonia, or central nervous system infection; respiratory distress syndrome can also be partly due to the preterm birth itself. It is reported that neonates with severe listeriosis supported with ECMO have a fair survival rate [31,32]. A vesicular or pustular rash is also described as a sign of Granulomatosis infantisepticum, a rare neonatal listeriosis condition characterized by skin multifocal granulomas [7,18,33].

Late-onset listeriosis occurred in 6% of babies belonging to the MONALISA cohort, and all developed meningitis: this unique pattern probably reflects a distinctive invasion mechanism, with ingestion of LM during passage through the birth canal [7]; these babies are usually born from asymptomatic mothers with negative blood cultures [18]. Listeria is the third leading cause of neonatal bacterial meningitis, after GBS and Escherichia Coli [34], with an overall incidence as high as 25% [20,24]. Overall, 10% of infected babies develop long-term neurological sequelae.

### 3.5. Treatment of LM Infection

Effective treatment of LM infection can be difficult because Listeria responds poorly to bacteriostatic antibiotics due to its intracellular nature and the formation of granulomatous tissue. Also, delayed diagnosis often defers treatment start. No specific guidelines are available, and there are no prospective in vivo studies in this area: therefore, recommendations are based on small case series or animal studies.

A crucial requirement for an effective antibiotic therapy is that the active substance penetrates into cells and maintains a high intracellular concentration, without causing significant alteration in the pH, which may reduce its effect. Also, in order to cause bacterial death, the antibiotic needs to bind to LM through bacterial penicillin-bound protein-3 (PBP3) [18,35]. Another obvious and critical characteristic is the ability to enter into the placenta in adequate concentration, since it is the main reservoir of infection [36], and to pass to the fetus.

The gold standard for LM therapy in pregnancy is high-dose Ampicillin (12 g/day), which was also used in our case; other options include Penicillin and Amoxicillin, both with lower effectiveness than Ampicillin [20,28,37,38,39,40,41]. Some in vitro studies suggest a synergistic effect of Gentamycin, which was not confirmed by studies on the animal model; the addition of Gentamycin is feasible, but not routinely recommended [36]. Cephalosporins and clindamycin are not useful in the treatment of LM infection [20]. In the general population, trimethoprim with sulfamethoxazole is considered the alternative treatment in case of penicillin allergy; however, this agent is an FDA category C drug and its use in pregnancy is not recommended, as it can cause heart defects and central nervous system anomalies linked to its antagonism with folic acid [10,20]. Unfortunately, cases of LM-acquired resistance to Tetracycline, Clindamycin, and Trimethoprim have been described [5,42]. The optimal duration of LM therapy in pregnancy has not been established, but some experts have suggested at least 3–4 weeks [36,43].

Antibiotic treatment seems effective in reducing maternal, fetal, and neonatal adverse outcomes [44,45]; favorable outcomes have also been reported for cases of LM infection occurring very early during pregnancy, after timely diagnosis, and immediate treatment [46]. Recently, encouraging results regarding the effectiveness of LM treatment in pregnancy have been reported by Charlier and colleagues: prenatal maternal antibiotic therapy resulted in being significantly associated with a reduction in the rate of infected newborns [40]. Also, maternal antibiotic therapy was associated with a reduced neonatal need for inotropic drugs, fluid resuscitation, and mechanical ventilation at birth [40]. A few case reports have also confirmed positive outcomes after antibiotic therapy in pregnancy [38,39,44,45].

Overall, these results suggest that in cases at high risk, Ampicillin treatment should be prescribed even in case of uncertain diagnosis, i.e., after consumption of contaminated food or in the presence of symptoms such as fever during labor, preterm labor, or uterine irritability, especially during an ongoing listeriosis outbreak [7,16,25,36,40].

## 4. Conclusions

LM infection in pregnancy is a rather infrequent, but potentially severe disease, which should be considered in the differential diagnosis in case of pregnant women presenting with fever and obstetric signs. It is associated with a significantly higher risk of fetal loss, preterm delivery, and adverse neonatal outcomes. The patient described in the case report presented with non-specific general symptoms such as fever associated with uterine contractions, which is the most frequent presentation of the disease. Blood cultures allowed for rapid diagnosis of the infection. Current literature demonstrates that timely diagnosis and appropriate antibiotic therapy are likely to be highly effective in improving fetal and neonatal prognosis. Indeed, in the presented case, the patient responded to therapy in an excellent way, and the infection was eradicated. Spontaneous labor, which is extremely frequent in twin pregnancies, was responsible for the preterm delivery of the twins. Both fetuses were born alive, did not contract LM, and are currently well: this allows us to conclude that diagnosis was timely and the established treatment was effective.
